# Paragangliomas and Anemia: Literature Review and Case Report

**DOI:** 10.3390/medicina59111925

**Published:** 2023-10-30

**Authors:** Maria-Daniela Tănăsescu, Ștefan Popescu, Alexandru Mincă, Teodora Isac, Emel Suliman, Maria Mihaela Grigorie, Emine Suliman, Daniel Stăniloaie, Delia Timofte, Dorin Ionescu

**Affiliations:** 1 Department 1 of Medical Semiology, Discipline of Medical Semiology and Nephrology, Bucharest Emergency University Hospital, Faculty of Medicine, ‘Carol Davila’ University of Medicine and Pharmacy, Dionisie Lupu Street, No. 37, Sector 2, 020021 Bucharest, Romania; maria.tanasescu@umfcd.ro (M.-D.T.); dorin.ionescu@umfcd.ro (D.I.); 2 Department of Nephrology, Bucharest Emergency University Hospital, 050098 Bucharest, Romania; stefanpopescutakurov@gmail.com; 3 Department 2 of Internal Medicine, Faculty of Medicine, ‘Carol Davila’ University of Medicine and Pharmacy, 020021 Bucharest, Romania; teodora.isac@drd.umfcd.ro; 4 Department 10 of General Surgery, Discipline of Surgery I, Bucharest Emergency University Hospital, Faculty of Medicine, ‘Carol Davila’ University of Medicine and Pharmacy, 020021 Bucharest, Romania; emel.suliman@umfcd.ro (E.S.); daniel.staniloaie@drd.umfcd.ro (D.S.); 5 Department 3 of Dentistry III, Discipline of Endodontics, Faculty of Dentistry, ‘Carol Davila’ University of Medicine and Pharmacy, 020021 Bucharest, Romania; 6 Department 3 of Complementary Sciences, Discipline of Medical Informatics and Biostatistics, Faculty of Medicine, ‘Carol Davila’ University of Medicine and Pharmacy, 020021 Bucharest, Romania; emine.suliman@umfcd.ro; 7 21st Department of General Surgery, Bucharest Emergency University Hospital, 050098 Bucharest, Romania; 8 Department of Dialysis, Bucharest Emergency University Hospital, 050098 Bucharest, Romania; delia.timofte@gmail.com

**Keywords:** benign tumor, carotid artery anastomosis, carotid body paraganglioma, chronic anemia, paraganglioma of head and neck

## Abstract

Paragangliomas are rare neuroendocrine tumors that arise from the extra-adrenal autonomic paraganglia, i.e., small organs consisting mainly of neuroendocrine cells that are derived from the embryonic neural crest and have the ability to secrete catecholamines. Paragangliomas can derive from either parasympathetic or sympathetic paraganglia. Most of the parasympathetic ganglia-derived paragangliomas are nonfunctional, and symptoms result from mass effect. Conversely, the sympathetic paragangliomas are functional and produce catecholamine. Although such patients could have symptoms similar to pheochromocytoma, mass effect symptoms, or non-specific symptoms, being benign tumors, they can also present with anemia, specifically iron-deficiency anemia. Considering that neoplastic pathology is chronically accompanied by moderate, normochromic, normocytic anemia, association between paragangliomas that are mostly benign but with a potential degree of malignancy and anemia is not as frequent as expected, with only 12 cases reported in the literature. We report a case of a 54-year-old female patient diagnosed with a paraganglioma of the carotid glomus accompanied by severe normochromic, normocytic anemia, which reached normal limits after excision of the paraganglioma.

## 1. Introduction

Paragangliomas are rare neuroendocrine tumors that arise from the extra-adrenal autonomic paraganglia, i.e., small organs consisting mainly of neuroendocrine cells that are derived from the embryonic neural crest and have the ability to secrete catecholamines. Paragangliomas are closely related to pheochromocytomas (which are sometimes referred to as intra-adrenal paragangliomas) and are histopathological indistinguishable, so the clinical diagnostic approach to differentiate between pheochromocytoma and paraganglioma is an important step considering the risk of malignancy and genetic testing. Due to the pandemic and reduced addressability to various health-care providers, such diagnostic strategies could be even more challenging [1].

Paragangliomas can derive from either parasympathetic or sympathetic paraganglia; the two types occur with similar frequency [2]. Parasympathetic and sympathetic paragangliomas differ in their anatomic distribution and frequency of an underlying genetic syndrome; they also have distinct clinical features.

The majority of parasympathetic ganglia-derived paragangliomas are located in the neck and skull base along the branches of the glossopharyngeal and vagus nerves. The most common locations are carotid body, jugulotympanic and vagal paraganglia, and rarely, laryngeal paraganglia. The majority of paragangliomas arising within the skull-base and neck region are not associated with catecholamine secretion; in various reports, up to 5% are symptomatic from hypersecretion [3,4,5]. About one-half of skull-base and neck paragangliomas arise in the setting of a known genetic syndrome [6,7].

Sympathetic paragangliomas arise outside of the adrenal gland anywhere along the sympathetic chain from the base of the skull (5%) to the bladder and prostate (10%) [6]. Approximately 75% of sympathetic paragangliomas arise in the abdomen, most often at the junction of the vena cava and the left renal vein, or at Zuckerkandl’s organ. About 10% arise in the thorax, including pericardial locations [8,9]. Sympathetic paragangliomas can also arise in the thyroid gland [10,11], adjacent to the thoracic spine [12], and at the level of the cauda equina [13]. The majority of paragangliomas arising outside of the skull base and neck, which are almost exclusively sympathetic and have excess catecholamine secretion (86% in one series [5]), almost always norepinephrine. This results in symptoms that are similar to those of an adrenal pheochromocytoma. Approximately 25% are part of a genetic syndrome [6].

Most paragangliomas appear to be sporadic [2]. In about 35% to 50% of cases [14,15], a paraganglioma is a component of an inherited syndrome [16,17,18,19]; the likelihood of an inherited syndrome is higher (about 40%) in children [20,21]. The majority of hereditary paragangliomas, particularly those arising in the skull base and neck, have been linked to pathogenic variants in the genes encoding different subunits of the succinate dehydrogenase (SDH) enzyme complex. In addition, susceptibility to pheochromocytomas and paragangliomas is established in multiple endocrine neoplasia types 2A and 2B (MEN2), neurofibromatosis type 1 (NF1), von Hippel Lindau (VHL) disease, and the Carney–Stratakis dyad. Most cases of hereditary paraganglioma are accounted for by pathogenic variants in SDHD, SDHB, and SDHC, VHL, and NF1 [20].

The majority of paragangliomas are benign. Malignant paragangliomas, as defined by metastatic behavior, are less common. An estimated incidence of malignant paraganglioma in the United States in 2002 was 93 cases per 400 million persons [2].

Most paragangliomas are diagnosed in the third to fifth decades. Results from one study revealed that mean age at diagnosis was 47 years [5]. However, patients with paragangliomas of the skull base and neck tend to be a little older at presentation compared with those with abdominal paragangliomas (43 versus 36 years of age in one study [22]). Patients with hereditary paragangliomas tend to develop disease about a decade earlier than do those with sporadic disease [20,23,24]. The male-to-female ratio is approximately equal among patients with hereditary paraganglioma, while sporadic tumors are much more common in females (71% versus 29%) [16]. 

Because the clinical patterns of paraganglioma are commonly described together with those of pheochromocytoma, the specific incidence of paraganglioma is largely unknown. The combined estimated annual incidence of pheochromocytoma/paraganglioma is approximately 0.8 per 100,000 person years [25], and there are approximately 500 to 1600 cases in the United States per year [26]. However, many such tumors remain undiagnosed during life because there are autopsy series that revealed a higher than expected prevalence [27,28].

Most of the parasympathetic ganglia-derived paragangliomas are nonfunctional (80% to 90%), and symptoms result from mass effect. Most sympathetic paragangliomas are functional and present with catecholamine hypersecretion (hypertension, episodic headache, sweating, and tachycardia [20]); a minority present with pain or other symptoms related to mass effect. Other less-common presentations include upper gastrointestinal hemorrhage [29], back or chest pain, cough, dyspnea or hoarseness, exercise-induced nausea and vomiting [30], or a presentation with metastatic disease (which most commonly involves the regional nodes, lung, bone, and liver) [31,32,33,34,35,36].

Although paragangliomas can have symptoms similar to pheochromocytoma, mass effect symptoms, or non-specific symptoms, being benign tumors, they can also present with anemia, specifically iron-deficiency anemia. This anemia occurs in about 30% of patients diagnosed with neoplasm [37] and is due to a low degree of chronic inflammation that accompanies oncological pathology [38]. Cancer-related anemia (CRA) is associated with elevated inflammatory markers, and it is characterized as a mild, normocytic normochromic anemia and a decreased circulating serum iron concentration and transferrin saturation despite ample iron stores (e.g., serum ferritin > 100 ng/mL). This suggests that the underlying mechanism of CRA is a defect in iron handling rather than a lack of iron per se. Such a condition is termed “functional iron deficiency” [37].

A strong correlation has been found between the prevalence and severity of anemia; the prevalence of increased mean plasma levels of inflammation markers including C-reactive protein (CRP), fibrinogen, interleukin (IL)-6, tumor necrosis factor (TNF)-α, IL-1β, erythrocyte sedimentation rate, ferritin, hepcidin, erythropoietin, and reactive oxygen species; and the stage of cancer [38,39,40]. Anemia may be a marker of persistent tumor activity since hemoglobin will rise after resection of paraganglioma and fall again with the appearance of metastases [41].

Regarding the association between paragangliomas and anemia, there are several cases described in the literature in patients of both sexes, aged between 9 and 67 years, and with multiple localizations, most frequently at the abdominal level (duodenum, stomach, and retroperitoneal), pelvic level, and thoracic level in the mediastinum but also at the head and neck level (jugulotympanic and jugular glomus level), associated in most cases with severe anemia but also in some cases with moderate anemia. No cases of paragangliomas presenting with mild anemia are described (see Table 1). 

## 2. Case Report

This case report is organized according to the CARE guideline. We present the case of a 54-year-old woman who presented to the emergency room with right hypochondral pain and fatigue. The patient was known with stage 1 hypertension ESC/ESH without additional risk factors, hemorrhoidal disease, and biliary lithiasis.

On clinical examination, a sclero-tegumentary pallor, positive Murphy’s sign, and a 2–3 cm diameter tumor formation not adherent to superficial planes, painless, and without signs of local inflammation was palpated at the left latero-cervical level. 

The blood analysis revealed severe hypochromic anemia, regenerative microcytic anemia (Hgb = 6.6 g/dL (reference range: 10.9–14.3 g/dL); MCV = 56.4 fL (reference range: 75.5–95.3 fL); MCHC = 30.9 g/dL (reference range: 32.3–35.6 g/dL); RET = 2.18% (reference range: 0.51–2.17%)), low blood iron value (Fe = 13 μg/dL (reference range: 50–160 μg/dL)), elevated ferritin (>500 ng/dL (reference range: 15–300 ng/dL)), C-reactive protein 162.4 mg/L (reference range: 0–5 mg/L), fibrinogen 750 mg/dL (reference range: 238–498 mg/dL), erythrocyte sedimentation rate 82 mm/h (reference range: 5–10 mm/h), and reactive thrombocytosis (457 × 10^3^/μL), and a test for occult hemorrhage with a positive result was targeted. Other parameters from the biochemistry panel were in normal range.

Given the low hemoglobin values and the positive occult hemorrhage test, the diagnosis of GI hemorrhage was considered. Upper digestive endoscopy revealed no bleeding. Lower digestive endoscopy was performed, and some internal hemorrhoids with erythematous surface were found, which could not explain the severe anemia. EnteroCT was performed, and there were found no changes suggestive of inflammatory or tumoral disease at the level of the digestive tract segments. Scintigraphy with 4 mg pyrophosphate-labelled hemogram with static acquisitions at 15 min, 150 min, and 24 h found inconclusive images for scintigraphically detectable hemorrhage. SPECT-CT images showed “webbed” hemorrhage at the level of the gastric antrum, which were refuted by a second upper GI endoscopy.

As the patient presented with pain in the right hypochondrium on superficial and deep palpation and a positive Murphy’s sign, an abdominal ultrasound was considered, which revealed a hyperechogenic formation with posterior shadow cone at the level of the gallbladder. 

As differential diagnosis for the latero-cervical tumor, we considered the following: inflammatory (viral, bacterial, or parasitic reactivated) or metastatic adenopathy, including other neuroendocrine pathologies (neuroendocrine carcinomas, Merkle cell carcinoma, and medullary thyroid carcinoma), schwannomas, vascular tumors and salivary tumors, lymphomas, and paragangliomas [54].

**Figure 1:**
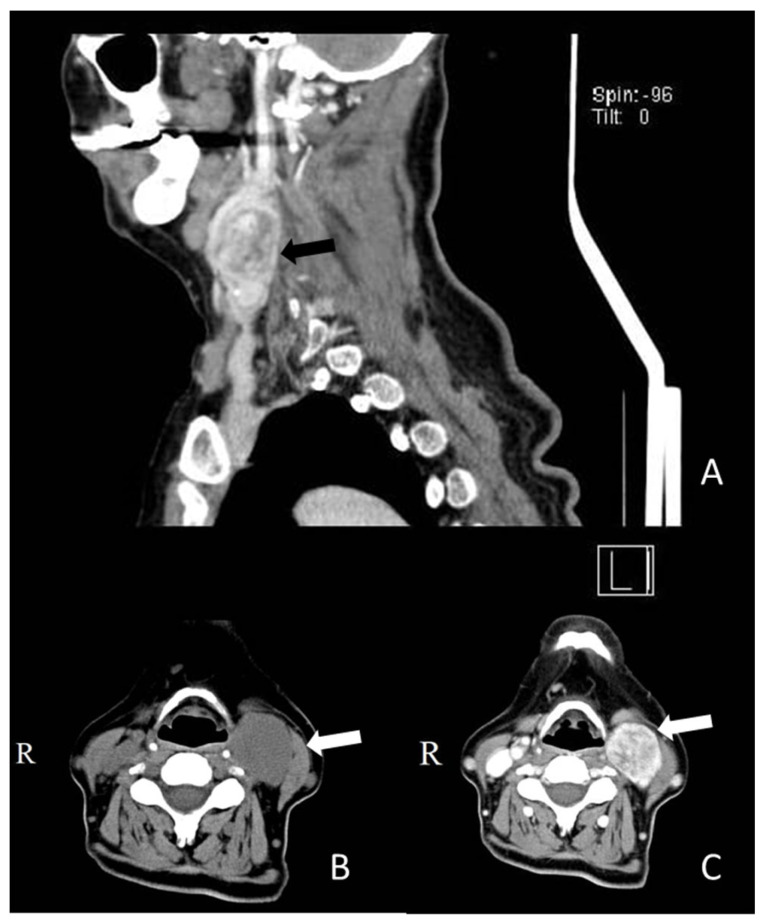
(**A**) CT scan of the left cervical region (sagittal section); the black arrow is pointing towards the tumoral mass. (**B**) CT scan of the left cervical region without contrast agent (transversal section); the white arrow is pointing towards the tumoral mass. (**C**) CT scan of the left cervical region with contrast agent (transversal section); the white arrow is pointing towards the tumoral mass.

In order to better appreciate the characteristics of the tumor formation at the left latero-cervical level, a CT scan of the cervical region was performed, which described a tissue expansion process located deeply latero-cervical and anterior to the jugulo-carotid bundle that it diverted; showing a segmental carotid artery sleeve with superior pole at the mandible angle adjacent to C3, C4, and C5 vertebrae; homogeneous and hypodense to adjacent musculature (SCM); with sharp, slightly irregular contour of 37/30 mm axial and 38 mm cranio-caudal dimensions. Iodophilia was intense and inhomogeneous, with multiple vascular tracts, late becoming hypodense, with appearance suggestive of carotid glomus (Figure 1). 

**Figure 2:**
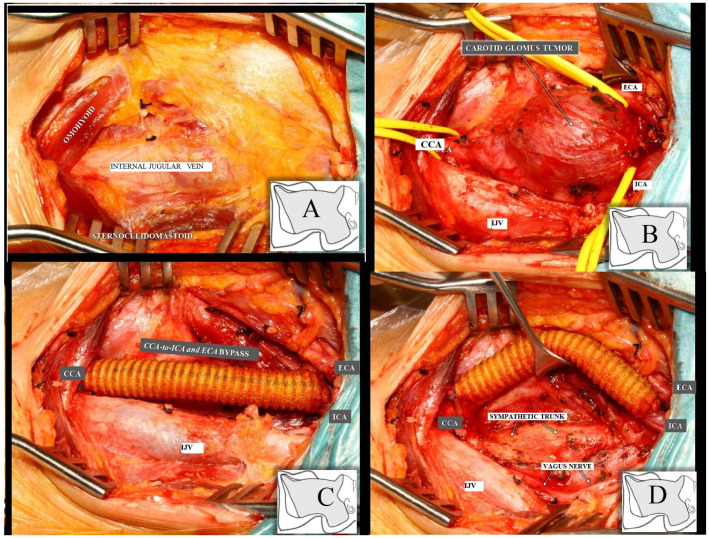
Intra-operatory stages: (**A**) After fascial resection. (**B**) Isolation of carotid glomus tumor. (**C**) After CCA-to-ICA and ECA BYPASS insertion. (**D**) Highlighting the neighborhood relationship of the CCA-to-ICA and ECA BYPASS with posterior anatomical structures.Abbreviation: CCA, common carotid artery; ECA, external carotid artery; ICA, internal carotid artery; IJV, internal jugular vein.

**Figure 3:**
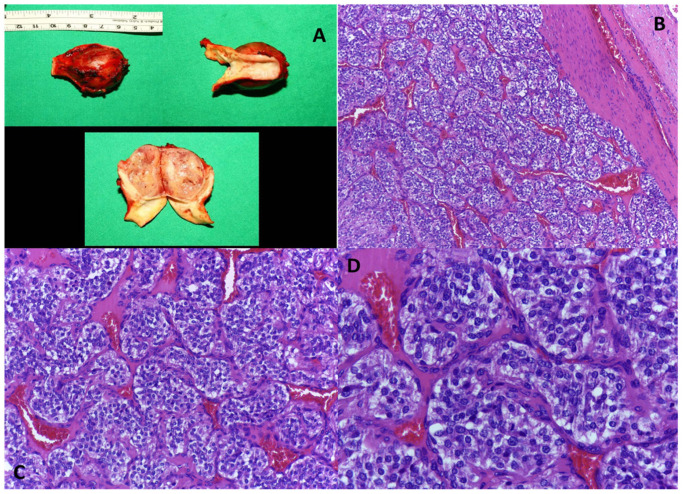
(**A**) Macroscopic aspect of carotid paraganglioma. (**B**) Paraganglioma, peripheral fibrous capsule, and nests of tumor cells separated by conjunctival-vascular septa; H.E. ob. 100×. (**C**) Paraganglioma, relatively regular nests of tumor cells separated by conjunctival-vascular septa; H.E. ob. 200×. (**D**) Paraganglioma, round tumor cells with central, regular nuclei and peripheral cytoplasm that is clear or pale eosinophilic and finely granular; H.E. ob. 400×.

Biopsy of the tumor was considered, and the patient was given two units of the isogroup isoRh erythrocyte mass (O (I),Rh+) to correct the level of hemoglobin concentration. Surgery was performed with general anesthesia and oro-tracheal intubation. Resection of the left cervical tumor was performed “en bloc” with the bifurcation of the left carotid artery (with which it formed a common body that could not be separated). The carotid arterial axis was restored by interposing a 6 mm Dacron prosthesis between the left common carotid artery and the left internal and external carotid arteries (Figure 2). Macroscopic examination described a greyish-white tissue fragment with a nodular appearance of 5/3.2/3 cm, showing a greyish color on section and relatively elastic consistency; the tumor formation appeared encapsulated and showed a slightly increased consistency in places (Figure 3A), and the microscopic examination described cell proliferation with organoid architecture (Zellballen-type structures) showing sustentacular cells at the periphery and main cells at the center; the stroma showed fibro-vascular structures. Uninvaded fibrous capsule and reactive-looking lymph node were also observed. The histopathological appearance described above is compatible with the diagnosis of carotid paraganglioma (Figure 3B–D) [55].

After excision of the tumor, the hemoglobin value reached normal limits and remained the same at the following medical re-evaluations. The patient was admitted postoperatively to the ICU and transferred to the nephrology department after 24 h. The patient recovered in 4 days and was discharged with the following recommendations: metoprolol 25 mg 1-0-0, pantoprazole 40 mg 1-0-1, folic acid 5 mg 1-0-0, and re-evaluation after 7 days. At the 7-day re-assessment, the patient’s general condition had significantly improved, the skin pallor had disappeared, the hemoglobin value had increased to 11 g/dL, and the cervical region was without local signs of inflammation. At the examination, which took place 30 days after discharge, the patient presented with the same clinical and paraclinical examination described above.

## 3. Discussion

Carotid body paraganglioma (CBP) is the most common paraganglioma of the head and neck, constituting 0.5% of all neoplastic pathologies encountered in the whole body [56]. This type of paragangliomas affects more women [54,56,57,58,59] and is bilateral in 10% of cases [60]. The involvement of hereditary factors is present in 4% to 9% of cases [54,56,57]. In 6–12% of cases, CBP can metastasize [54,57,58,61], a feature that can be identified, in the absence of a histopathological finding [62], in loco-regional lymph nodes, or in rarer cases, there may be distant metastases [16].

The optimal approach to therapy depends on the symptomatology, size, location, the relationship between the tumor and neurovascular structures as well as the age and general health of the patient [63,64,65,66,67,68,69].

The variable growth rate, along with the fact that the majority (90% or more) are nonfunctioning, is an important consideration in determining whether to intervene therapeutically versus pursue an initial period of surveillance. Initial observation is an acceptable approach for individual patients who have small (e.g., <1 cm), asymptomatic, non-secreting jugular and carotid body paragangliomas that can be closely monitored to assess the natural history [70]. The British Skull Base Society advocates for an initial period of surveillance for many patients with skull-base and neck paragangliomas, with the following exceptions [71]: tympanic paragangliomas, jugular paragangliomas with troublesome conductive hearing loss, pulsatile tinnitus, impending or actual facial nerve weakness, significant brainstem compression, secretory tumors, cranial nerve dysfunction related to tumor burden, clinical evidence of rapid growth, malignant disease, or patient choice.

The benign nature of this type of paraganglioma is illustrated in several studies. Two of these studies showed that the time of doubling of the tumor size was between 4.2 and 13.8 years [72,73]. Another study mentioned that for an average duration of 4 years, during which the patients were followed-up, the average annual growth rate was 1 mm (range between 0.3 and 5 mm per year), and the average time of doubling of the tumor size was 4 years (range between 0.6 and 21.5 years) [74]. A fourth study, over an average of 5 years of patient follow-up, showed that 42% of the tumors analyzed maintained their size, 38% changed their size by an average of 2 mm per year, and 20% reduced their size [75]. 

For symptomatic CBP or size greater than 2 cm, complete surgical resection is indicated, which historically has been the therapeutic method of choice for cervical paragangliomas, as for our patient [62,65,66,75]. The cure rate after excision of benign CBP is between 89% and 100% [66,76,77,78,79]. The cure rate is lower in patients with loco-regional lymphatic metastases (in a National Cancer Database series, the 5-year survival rate was 77% [60]. 

Our patient presented no significant postoperative morbidity. However, the major morbidity associated with surgery, as revealed by the literature, is related to postoperative cranial nerve dysfunction [31,63,66,67,72,80,81,82]. In a retrospective, multicenter study carried out over 26 years, the risk of stroke, bleeding, or cranial nerve dysfunction was specified in 1%, 6%, and 19% of cases, respectively [63]. 

Primary radio therapy (RT) is a reasonable alternative to surgery, particularly if resection would require sacrifice of critical vascular and/or neural structures, for those with recurrent tumor after previous surgery, and in the setting of a contralateral vagal nerve palsy. When defined in this way, at doses between 45 and 56 Gy, long-term tumor control rates for carotid body paragangliomas are 90 to 96% [77,83,84,85,86]. Permanent cranial nerve deficits seem to be less common after RT of cervical paragangliomas when compared with results of surgical resection [72,73,77,81,86]. Using a multidisciplinary approach, we decided, however, for our patient, on a primary surgical strategy.

Multiple tumors occur in up to one-third of cases [87]. Resection of bilateral carotid body tumors may cause baroreflex failure syndrome, which is characterized by severe, constant hypertension in the first 24 to 72 h after surgery, followed by labile hypertension and hypotension, headaches, emotional instability, and palpitations [88,89]. For patients with bilateral carotid body paragangliomas, surgical excision of the smaller tumor should be done first; if the vagus and hypoglossal nerves are functional, contralateral surgery may be performed [87]. Conversely, in the case of injury of these nerves, no contralateral tumor excision should be performed given that bilateral neurological deficits with dramatic consequences might occur. In these cases, RT is appropriate for the opposite tumor.

Patients with multiple tumors, metastatic disease, or an identified genetic pathogenic variant should have long-term radiologic and clinical follow-up for local recurrence or for development of paragangliomas outside of head and neck [71]. In a study, in 35% of patients’ synchronous metastasis was identified, and in 65% of patients, metastasis developed in a median of 5.5 years (range between 0.3 and 53.4 years) from initial diagnosis [90]. A significant proportion of patients with apparently benign paraganglioma (31% [5]) will have persistent or recurrent disease or develop metachronous primary tumor(s). The interval between treatment of a primary tumor and appearance of a recurrence or metastasis may be long, with some recurrences reported up to 53 years after the initial diagnosis [77,90,91,92,93,94,95]. With routine long-term follow-up, the malignancy rate has been higher than suspected previously (16% at 10 years in one report of combined pheochromocytoma/secretory paraganglioma) [96]. The National Comprehensive Cancer Network [97] suggests monitoring blood pressure and biochemical markers every 6 to 12 months for the first three years after resection, then annually to year 10, and obtaining imaging studies only as clinically indicated. For patients with locally unresectable disease or distant metastases, the guidelines recommend monitoring blood pressure and biochemical markers every three to four months and imaging as clinically indicated.

This case report highlights the diagnostic evaluation, treatment approach, and follow-up of a patient with a carotid body tumor and anemia, with the paraganglioma being discovered by chance on objective and paraclinical examination. The typical feature of all the cases mentioned above is that paragangliomas may be accompanied by moderate or severe anemia, an association which was also found in the case presented by us. Also, the hypothesis that after paraganglioma resection, the hemoglobin value increases, reaching normal limits, is tested with a positive result in the present case.

## 4. Conclusions

In conclusion, even if paragangliomas are rare and benign tumors, they can cause severe anemia with systemic manifestations, which can be improved by diagnosing the cause and applying appropriate treatment using a multidisciplinary approach.

## Figures and Tables

**Table 1:** Summary of clinical cases with paraganglioma and anemia, Abbreviation: F, female; M, male; GIST, gastrointestinal stromal tumor. Moderate anemia corresponds to a level of hemoglobin concentration of 7.0–9.9 g/dL, while severe anemia corresponds to a level less than 7.0 g/dL.

CRT. No.	Gender	Age	Location of Paraganglioma	Severity of Anemia	Type of Tumor	Metastases	Associated Pathology
1 [42]	F	-	Glomus jugulare	Severe	Malignant	Lung	-
2 [43]	M	27	Retroperitoneal	Severe	Benign	-	Hemoperitoneum
3 [44]	M	17	Mediastinal	Severe	Benign	-	-
4 [45]	M	-	Mediastinal	Moderate	Benign	-	GIST
5 [46]	F	67	2nd portion of duodenum	Moderate	Malignant	Lymph nodes	-
6 [47]	F	62	1st portion of duodenum	Severe	Benign	-	-
7 [48]	M	38	1st portion of duodenum	Severe	Benign	-	-
8 [49]	M	17	Pelvic	Moderate	Benign	-	IL-6 secreting paraganglioma mimicking multicentric Castleman disease
9 [50]	F	9	Gastric	Severe	Benign	-	GIST
10 [51]	M	-	Jugulotympanic	Severe	Malignant	Extent to nasopharynx	-
11 [52]	F	19	4th portion of duodenum	Severe	Malignant	Pelvic	-
12 [53]	F	39	Hepatic	Moderate	Benign	-	-

## Data Availability

The data presented in this study are available on request from the corresponding author. The data are not publicly available due to privacy restrictions.
